# Supplementary material for: Amyloid-β oligomers are captured by the DNAJB6 chaperone: Direct detection of interactions that can prevent primary nucleation
Source: J Biol Chem. 2020 Apr 29;295(24):8135–44. doi: 10.1074/jbc.RA120.013459 (PMC7294096; doi:10.1074/jbc.RA120.013459)
Supplement: Supporting Information [file supp_295_24_8135__index.html]

Amyloid-β oligomers are captured by the DNAJB6 chaperone: Direct detection of interactions that can prevent primary nucleation — Aβ oligomers captured by DNAJB6 — Amyloid-β oligomers are captured by the DNAJB6 chaperone: Direct detection of interactions that can prevent primary nucleation — EDITORS' PICK: Aβ oligomers captured by DNAJB6 — Supporting Information 

# Amyloid-β oligomers are captured by the DNAJB6 chaperone: Direct detection of interactions that can prevent primary nucleation

## Supporting Information

- Supporting Information (to be published online) - Supporting Information Fig. S1-S9
