# Supplementary material for: Amyloid-β oligomers are captured by the DNAJB6 chaperone: Direct detection of interactions that can prevent primary nucleation
Source: J Biol Chem. 2020 Apr 29;295(24):8135–44. doi: 10.1074/jbc.RA120.013459 (PMC7294096; doi:10.1074/jbc.RA120.013459)
Supplement: Supporting Information [file supp_RA120.013459_159503_2_supp_517103_q98wqy.pdf]

SUPPORTING INFORMATION FOR

Amyloid-β oligomers are captured by the DNAJB6 chaperone: Direct detection of interactions that can prevent primary nucleation

Nicklas Österlund[1], Martin Lundqvist[2], Leopold L. Ilag[3], Astrid Gräslund[1], Cecilia Emanuelsson[2]

[1]Department of Biochemistry and Biophysics, Stockholm University, Stockholm, Sweden, [2]Department of Biochemistry and Structural Biology, Lund University, Sweden, [3]Department of Environmental Science and Analytical Chemistry, Stockholm University, Sweden.

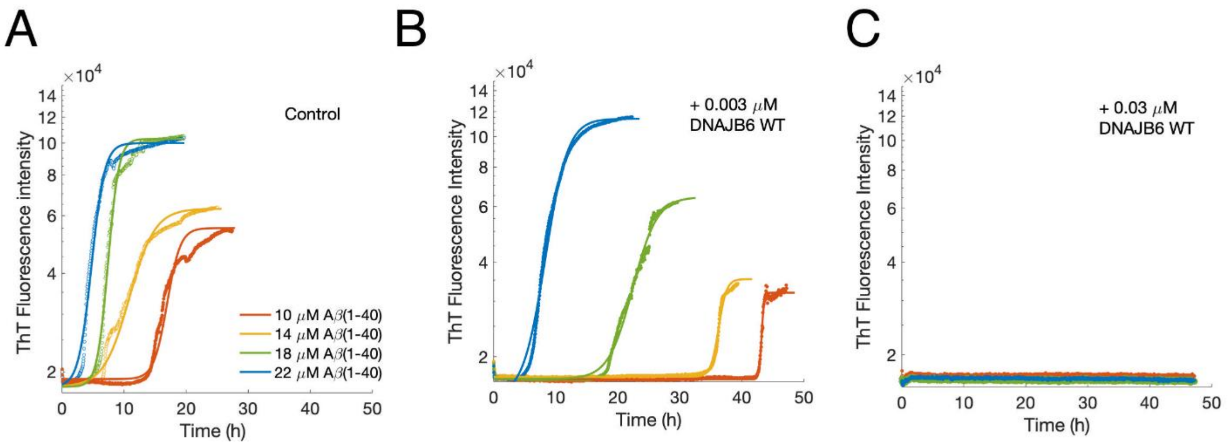

Figure S1. The capacity of DNAJB6 to suppress fibril formation determined by time-dependent ThT fluorescence increase. (A) Aβ(1-40) at concentrations 10-22 μM, **(B)** Same Aβ(1-40) concentrations as in (A) and 3 nM DNAJB6 WT, **(C)** Same Aβ(1-40) concentrations as in (A) and 30 nM DNAJB6 WT.

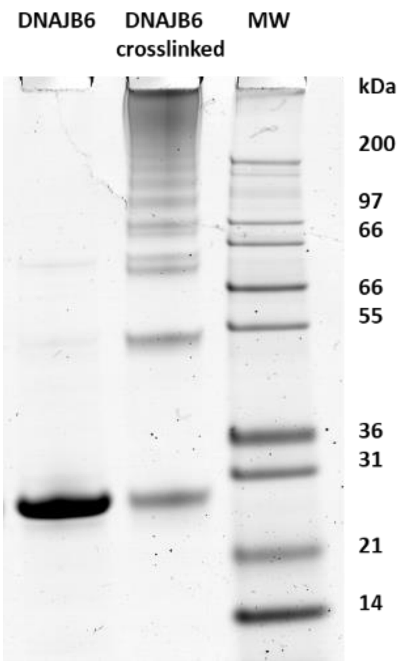

Figure S2. DNAJB6 for measurements of suppression of fibril formation. DNAJB6 oligomers subjected to denaturing electrophoresis without or with prior crosslinking at a DNAJB6 protein concentration of 50 µM in a buffer with 20 mM sodium phosphate pH 8.0, 150 mM NaCl and the crosslinker BS3 (bis(sulfosuccinimidyl)suberate) added at 3 mM final concentration.

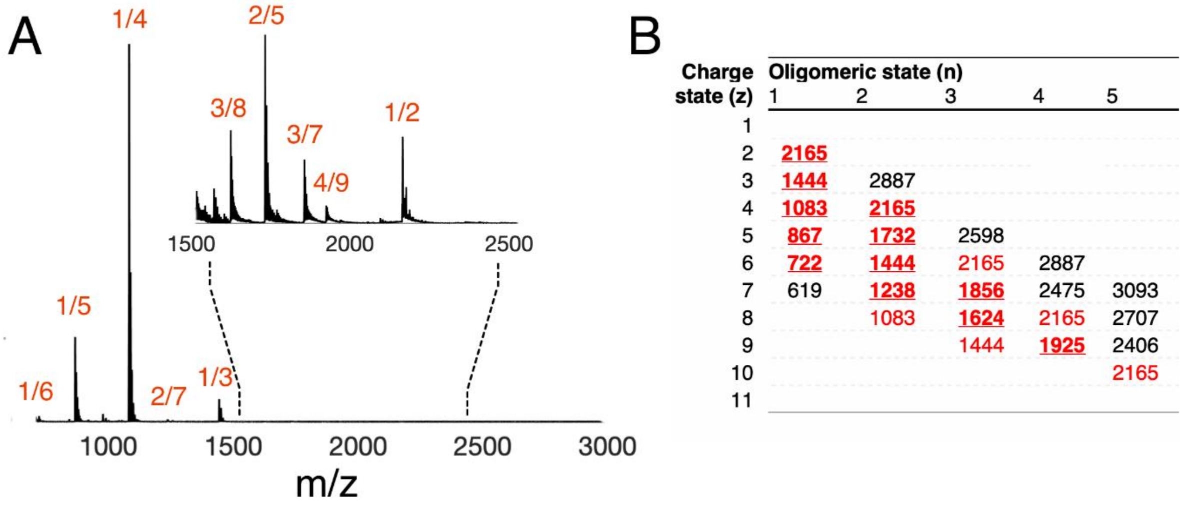

B

Charge state (z)	Oligomeric state (n) 1	2	3	4	5
1					
2	**2165**				
3	**1444**	2887			
4	**1083**	**2165**			
5	**867**	**1732**	2598		
6	**722**	**1444**	2165	2887	
7	619	**1238**	**1856**	2475	3093
8		1083	**1624**	2165	2707
9			1444	**1925**	2406
10					2165
11					

Figure S3. (A) Mass Spectrum of recombinant Aβ(1-40) under native conditions. The red numbers indicate the oligomeric state/charge state ratio (n/z) for each peak. The inset shows a magnification of the part of the spectrum where the oligomeric species are found. **(B)** Table with theoretical m/z values for Aβ(1-40) ions. The red numbers are the m/z values which are experimentally observed. Red numbers which are bold and underlined represent n/z states which have been confidently assigned based on isotopic pattern and/or ion mobility drift times.

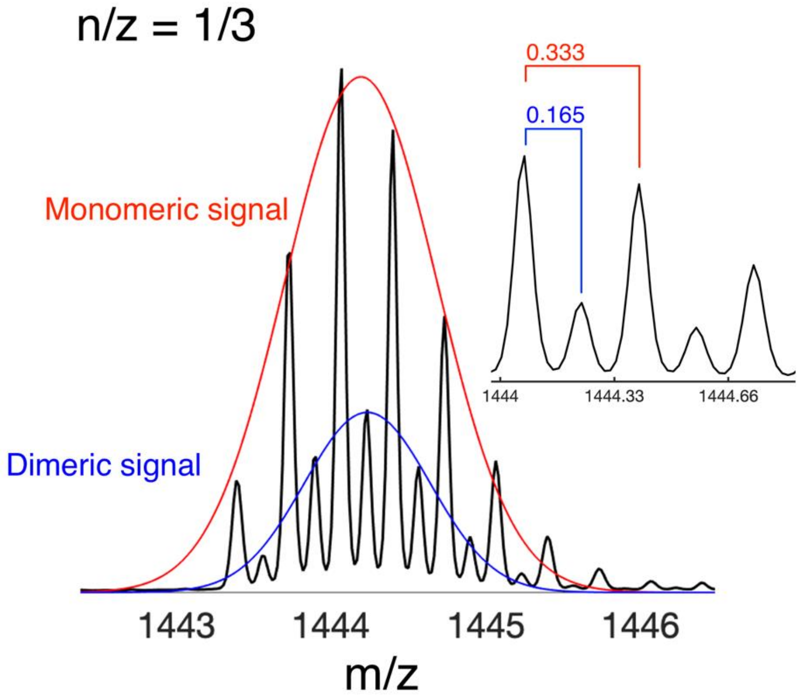

Figure S4. Overlapping n/z states can be deconvoluted in the m/z dimension based on the ^{13}C isotopic pattern. The distance between isotopic peaks is 1/z. The n/z peak is shown and the two oligomeric states have been fitted using a Gaussian peak shape.

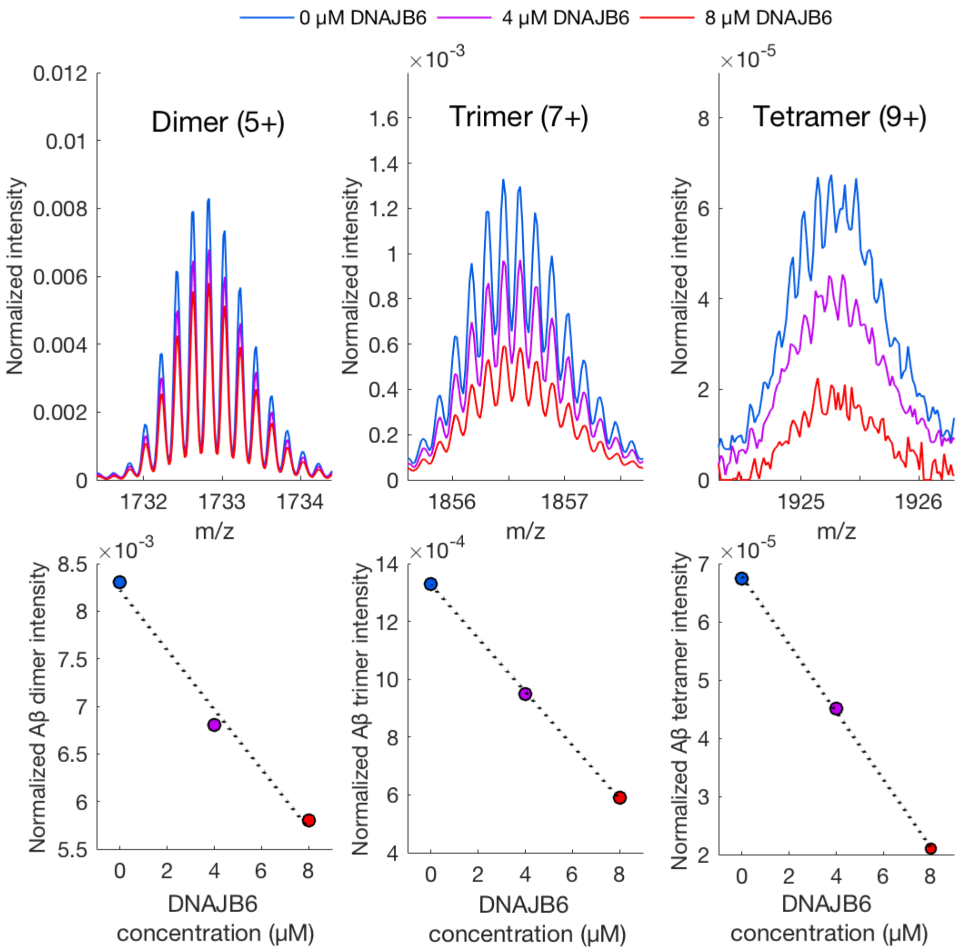

Figure S5. Detected native mass spectrometry signals from oligomeric states (dimers, trimers and tetramers) of Aβ(1-40) formed during pre-incubation in solution of 30 μM Aβ(1-40) in 10 mM ammonium acetate pH 7, without or with 8 and 4 μM DNAJB6 corresponding to molar ratio Aβ(1-40) to DNAJB6 1:0.13 and 1:0.066, respectively. DNAJB6 decreases the detectable amount of Aβ(1-40) oligomers in a concentration-dependent manner and the removal of Aβ(1-40) oligomers from solution is increasingly more efficient for dimers-trimers-tetramers. Data presented in mass spectra show signals from one measurement. Each measurement was repeated in at least three independent experiments on different date, with 1 h incubation of Aβ(1-40) without or with DNAJB6, with similar results.

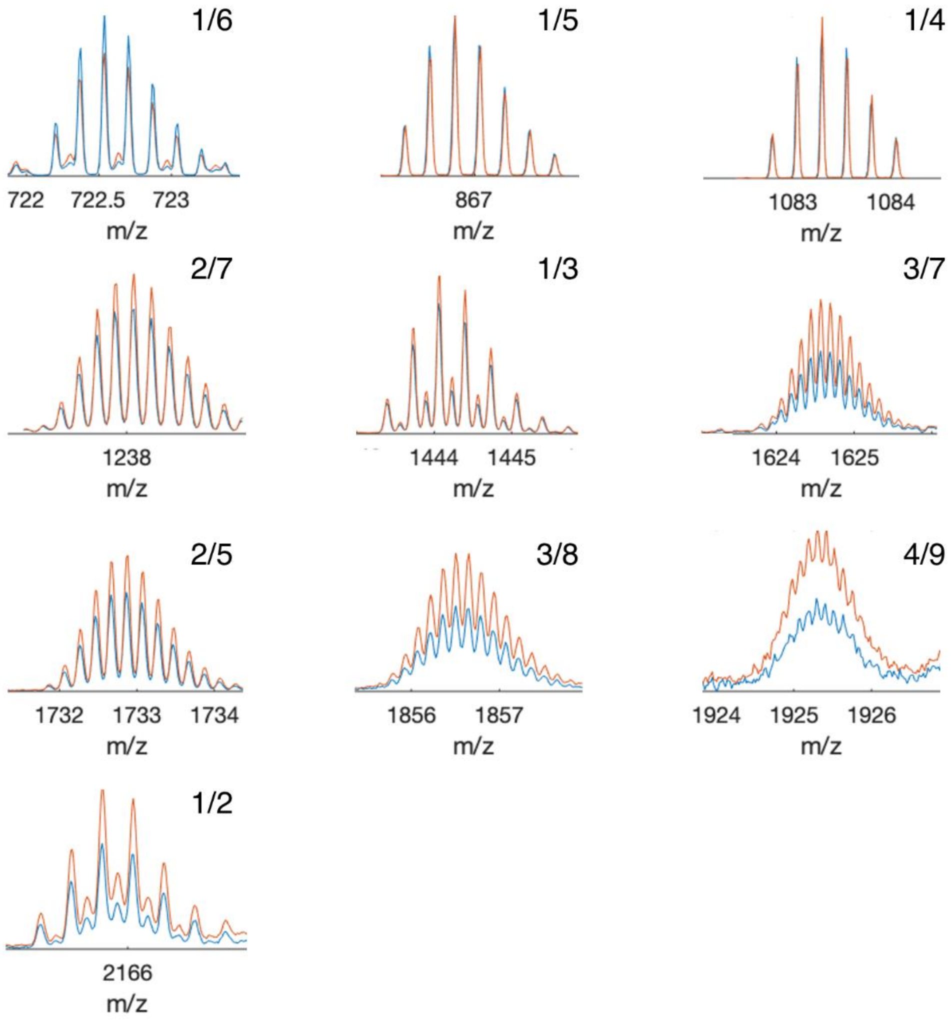

Figure S6. Individual n/z signals for Aβ(1-40) at 0h (blue) and after 1h (red) of pre-incubation at 37 °C. Normalized intensities are shown (intensity of the specific peak/total intensity of all peaks in the mass spectrum of that peak). The oligomeric components increase relative to the monomeric component during the 1 h incubation.

Figure S6.

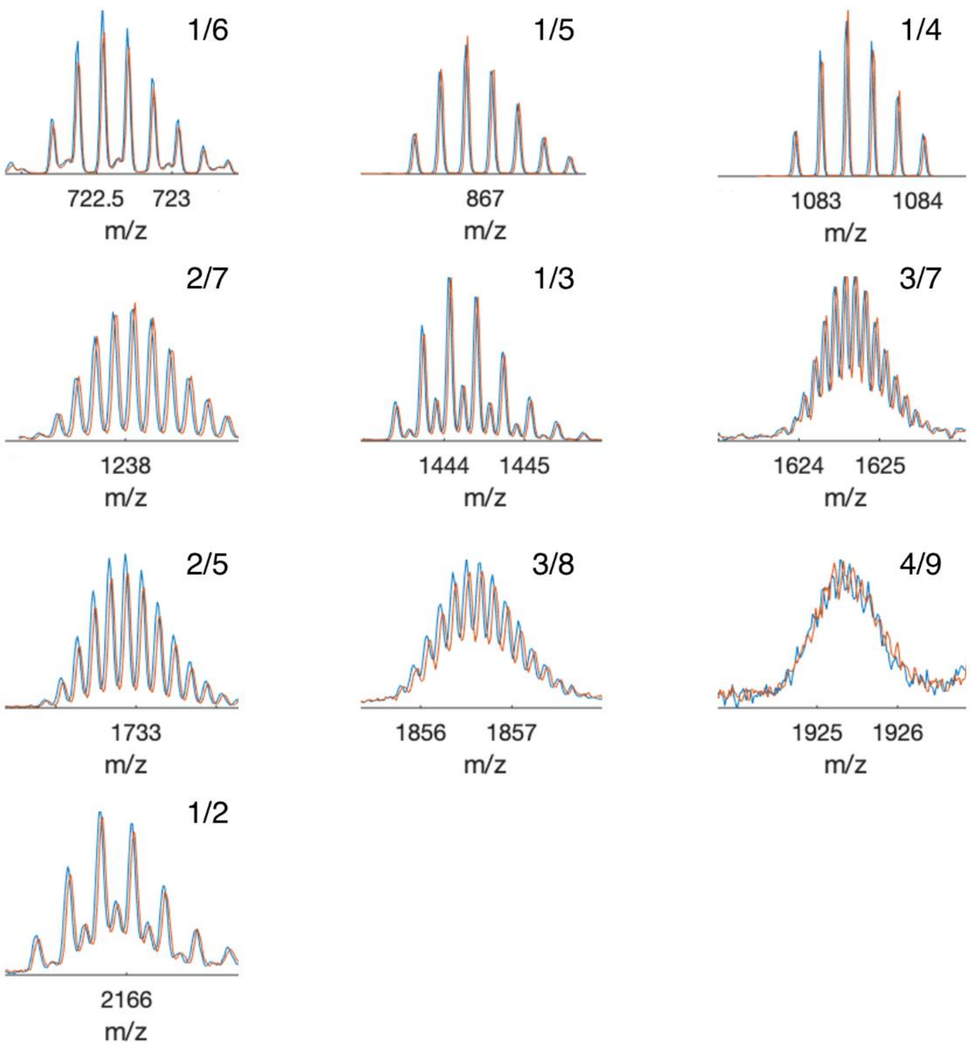

Figure S7. Individual n/z signals for Aβ(1-40) at 0h (blue) and after 1h (red) of pre-incubation at 37 °C together with DNAJB6 WT. Normalized intensities are shown (intensity of the specific peak/total intensity of all peaks in the mass spectrum of that peak). Compared to Fig. S6 where the oligomeric components increase relative to the monomeric component during the 1 h incubation the presence of DNAJB6 inhibits the increase of oligomeric components relative to the monomeric component.

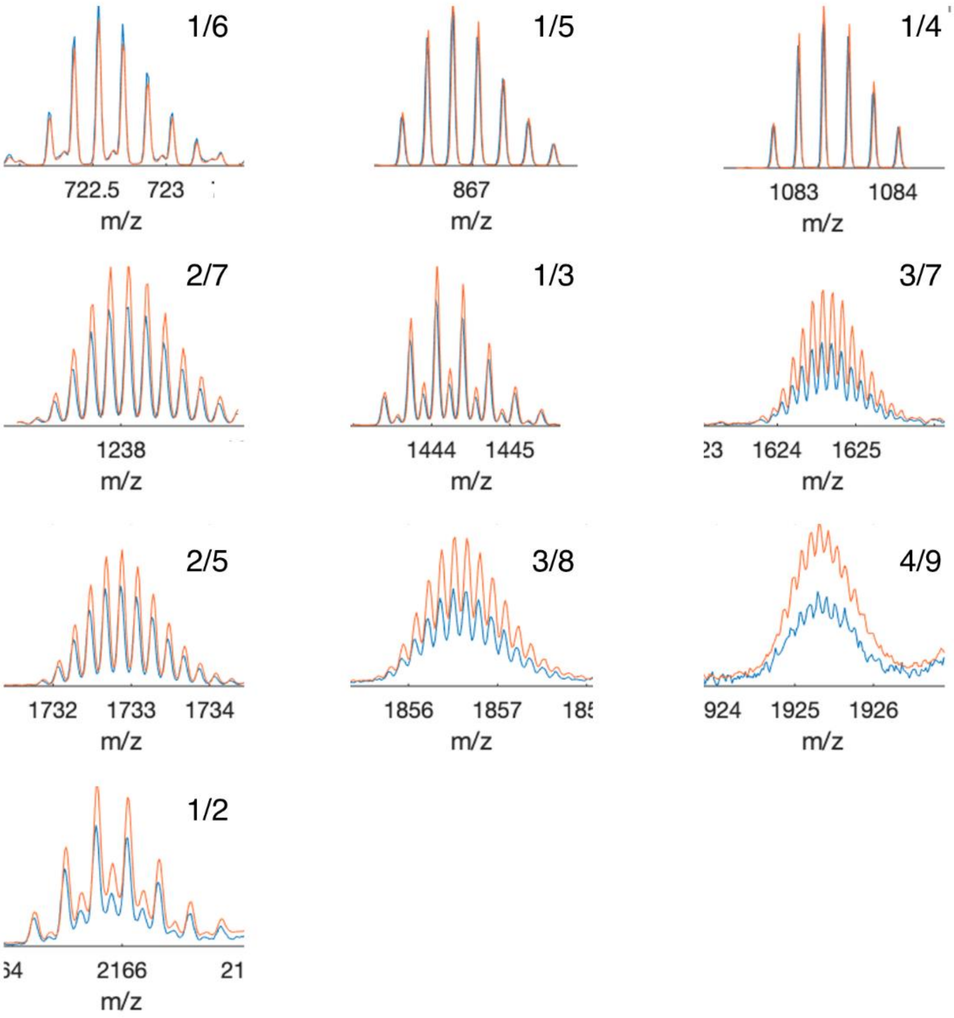

Figure S8. Individual n/z signals for Aβ(1-40) at 0h (blue) and after 1h (red) of pre-incubation at 37 °C together with DNAJB6 S/T18A. Normalized intensities are shown (intensity of the specific peak/total intensity of all peaks in the mass spectrum of that peak). Compared to Fig. S6 where the the presence of DNAJB6 WT inhibits the increase of oligomeric components relative to the monomeric component the S/T18A mutant does not inhibit and the oligomeric components increase relative to the monomeric component during the 1 h incubation, resembling the situation in Fig. S6.

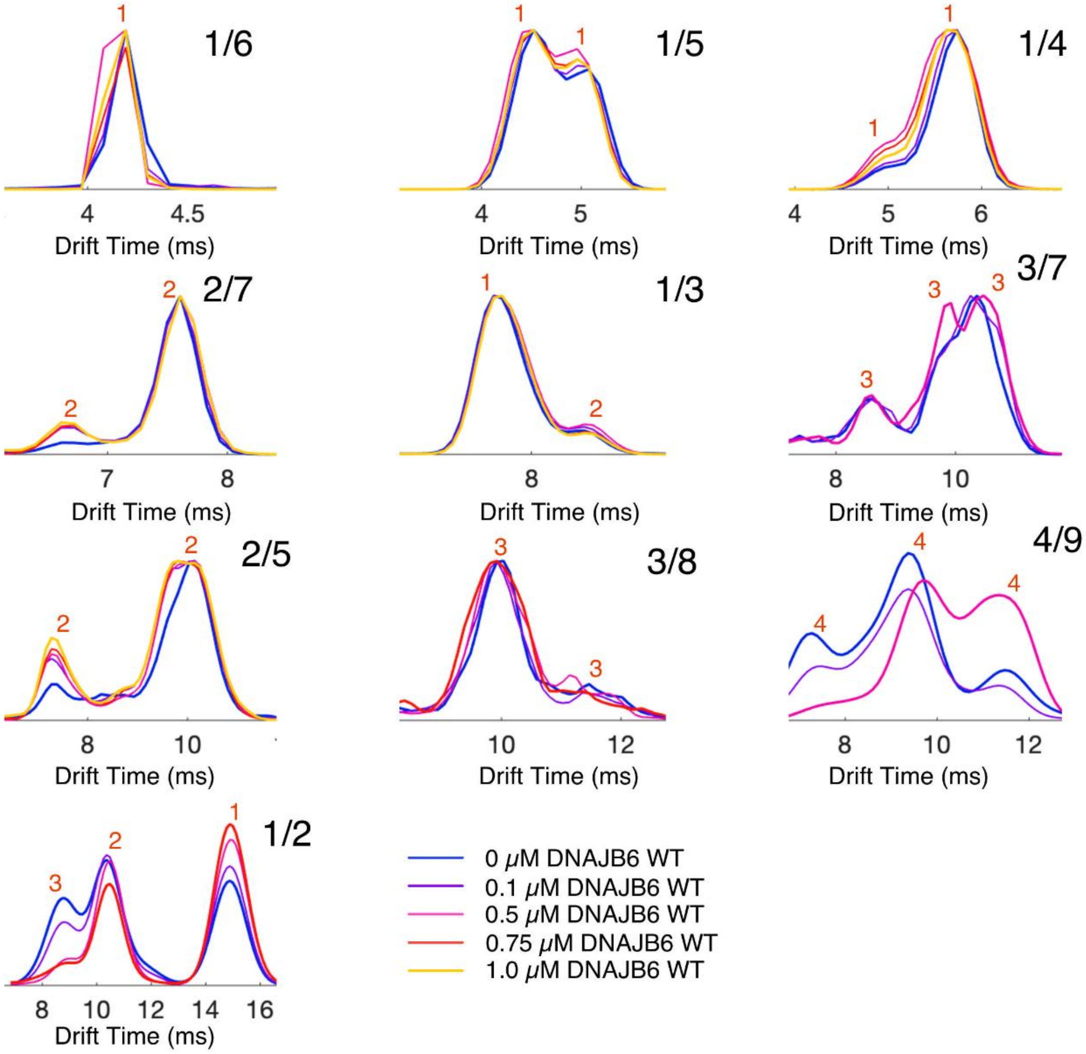

Figure S9. Ion mobility drift profile of the individual Aβ(1-40) n/z states after pre-incubation with different concentrations of DNAJB6 WT. The small red numbers indicate the oligomeric state of the drift time peak (based on isotopic pattern in the m/z dimension). It can be seen that some monomeric and oligomeric charge states exhibit several gas phase conformations. Incubation with DNAJB6 changes the relative occupancy of these conformational states in some cases, with shifts towards both more compact (shorter drift time, e.g. n/z = 2/7, 2/5) and more extended (longer drift time, e.g. n/z = 4/9) states.
